# Detection of haematuria and proteinuria reflects injury to significantly different glomerular surface areas: a quantitative hypothesis

**DOI:** 10.1093/ckj/sfaf245

**Published:** 2025-07-31

**Authors:** Osamu Uemura

**Affiliations:** Ichinomiya Medical Treatment and Habilitation Center, Ichinomiya, Aichi, Japan

To the Editor,

We propose a quantitative hypothesis to explain why haematuria and proteinuria detected by dipstick represent glomerular injuries of vastly different surface area extents.

Passage of blood components into the urinary space is thought to require full-thickness disruption of the three-layer glomerular filtration barrier—endothelium, glomerular basement membrane (GBM) and podocytes [1]. Using simple clinical parameters, we estimate the effective damaged surface area necessary for each urinary abnormality in a typical 10-year-old child with a daily glomerular filtered volume of 100 l and a urine output of 1 l (i.e. 100-fold urinary concentration).

For 1+ proteinuria (30 mg/dl), this corresponds to ≈0.3 mg/dl of filtered protein. Given a normal serum protein concentration of 7.0 g/dl (70 000 mg/dl), this implies injury to ≈0.004% of the glomerular filtration surface. Conversely, for 1+ haematuria [20 red blood cells (RBCs)/μl], the reverse-concentrated filtrate would contain 0.2 RBCs/μl. With a peripheral blood RBC count of 5 000 000/μl, this equates to injury over only 0.000004% of the filtration surface—indicating an ≈1000-fold sensitivity difference between proteinuria and haematuria. This distinction supports epidemiological findings that proteinuria is a stronger predictor of progressive glomerular injury and end-stage renal disease [2].

These calculations also help interpret renal biopsy findings. At the 1+ level, proteinuria may yield normal light microscopy findings [1]. When proteinuria increases to 2+ (≈100 mg/dl), the affected surface area increases to ≈0.01%, assuming a linear relationship. Meanwhile, haematuria-only patients often have normal biopsy results because the damaged area may be too small to be captured by a standard biopsy sample.

The sampling limitation is considerable. The human kidney contains ≈2 million glomeruli (1 million per kidney), yet a standard renal biopsy typically includes ≈20 glomeruli, only ≈0.001% of the total glomerular population. Hence small, focal injuries may go undetected unless they surpass this threshold (Table 1).

**Table 1:** Estimated glomerular surface area required for detection of urinary abnormalities.

Urinary dipstick	Urinary threshold	Estimated affected surface area (%)
1+ haematuria	≥20 RBCs/μl	0.000004
1+ proteinuria	≥30 mg/dl	0.004
2+ proteinuria	≥100 mg/dl	0.01
Kidney biopsy (≈20 glomeruli/2 million)		0.001

Our model is consistent with the clinical course of Alport syndrome, where haematuria often precedes proteinuria by several years [3, 4]. It may also explain why early immunoglobulin A nephropathy—initially localized in the mesangium—remains undetectable until inflammation extends to adjacent capillary loops and the GBM [5]. It is important to clarify that the estimated surface area required for detection reflects the threshold sensitivity of urinalysis findings rather than the temporal sequence or severity of glomerular disease progression. For example, in Alport syndrome, haematuria may appear earlier than proteinuria not because the injury is more extensive, but because a tiny focal full-thickness GBM disruption (≈0.000004% of the surface area) can cause detectable haematuria. In contrast, proteinuria becomes apparent only after damage exceeds the ≈0.004% threshold. Thus our model is fully consistent with the clinical observation of haematuria preceding proteinuria in Alport syndrome.

We acknowledge that this model simplifies the complex physiology of protein and RBC handling. While low molecular weight proteins are efficiently reabsorbed in the proximal tubule, albumin—being a high molecular weight protein—is minimally reabsorbed under physiological conditions. Thus detectable albuminuria largely reflects glomerular filtration that exceeds this limited tubular reabsorption capacity. Regarding haematuria, RBCs may lyse in hypertonic urine, potentially reducing RBC counts on microscopy. However, dipstick urinalysis detects haemoglobin itself, whether within intact erythrocytes or lysed, and is not significantly affected by red cell lysis. Therefore, our model—based on dipstick detection thresholds—remains valid despite these downstream modifications in the urinary tract.

However, we acknowledge important limitations. This model does not apply to sclerotic or fully scarred glomeruli, which do not filter and thus do not contribute to urinary findings. In addition, charge-selective injury may lead to proteinuria without structural disruption of the filtration barrier surface. Therefore, our model is applicable primarily to structural disruptions involving all three layers.

Despite its simplifications, this quantitative framework offers a novel physiological rationale to interpret urinalysis findings and better understand the threshold for glomerular injury detection. Future studies correlating these theoretical estimates with clinical and histological data would be valuable in validating this model.
